# Electroacoustic verification of frequency modulation systems in cochlear implant users^☆^

**DOI:** 10.1016/j.bjorl.2017.11.001

**Published:** 2017-12-26

**Authors:** Vanessa Luisa Destro Fidêncio, Regina Tangerino de Souza Jacob, Liége Franzini Tanamati, Érika Cristina Bucuvic, Adriane Lima Mortari Moret

**Affiliations:** a Universidade de São Paulo (USP), Faculdade de Odontologia de Bauru (FOB), Departamento de Fonoaudiologia, Bauru, SP, Brazil; b Universidade de São Paulo (USP), Hospital de Reabilitação de Anomalias Crânio-Faciais (HRAC), Bauru, SP, Brazil

**Keywords:** Cochlear implant, Assistive technology, Speech perception, Pediatric, Implante coclear, Tecnologia assistiva, Percepção da fala, Pediatria

## Abstract

**Introduction:**

The frequency modulation system is a device that helps to improve speech perception in noise and is considered the most beneficial approach to improve speech recognition in noise in cochlear implant users. According to guidelines, there is a need to perform a check before fitting the frequency modulation system. Although there are recommendations regarding the behavioral tests that should be performed at the fitting of the frequency modulation system to cochlear implant users, there are no published recommendations regarding the electroacoustic test that should be performed.

**Objective:**

Perform and determine the validity of an electroacoustic verification test for frequency modulation systems coupled to different cochlear implant speech processors.

**Methods:**

The sample included 40 participants between 5 and 18 year's users of four different models of speech processors. For the electroacoustic evaluation, we used the Audioscan Verifit device with the HA-1 coupler and the listening check devices corresponding to each speech processor model. In cases where the transparency was not achieved, a modification was made in the frequency modulation gain adjustment and we used the Brazilian version of the “Phrases in Noise Test” to evaluate the speech perception in competitive noise.

**Results:**

It was observed that there was transparency between the frequency modulation system and the cochlear implant in 85% of the participants evaluated. After adjusting the gain of the frequency modulation receiver in the other participants, the devices showed transparency when the electroacoustic verification test was repeated. It was also observed that patients demonstrated better performance in speech perception in noise after a new adjustment, that is, in these cases; the electroacoustic transparency caused behavioral transparency.

**Conclusion:**

The electroacoustic evaluation protocol suggested was effective in evaluation of transparency between the frequency modulation system and the cochlear implant. Performing the adjustment of the speech processor and the frequency modulation system gain are essential when fitting this device.

## Resumo

**Introdução:**

O sistema de frequência modulada é um dispositivo que ajuda a melhorar a percepção da fala no ruído, e é considerado a abordagem mais benéfica para melhorar o reconhecimento da fala no ruído em usuários de implantes cocleares. De acordo com as diretrizes, é necessário realizar uma verificação no momento da adaptação do sistema de frequência modulada. Embora existam recomendações sobre os testes comportamentais a serem realizados na adaptação do sistema de frequência modulada para usuários de implantes cocleares, não há recomendações publicadas sobre o teste eletroacústico que deve ser realizado.

**Objetivo:**

Realizar e validar um teste de verificação eletroacústica para sistemas de frequência modulada acoplados a diferentes processadores de fala de implante coclear.

**Método:**

A amostra incluiu 40 participantes com idades entre cinco e 18 anos, usuários de quatro modelos de processadores de fala. Para a avaliação eletroacústica, utilizou-se o dispositivo Audioscan Verifit com o acoplador HA-1 e os dispositivos de verificação de escuta correspondentes a cada modelo de processador de fala. Nos casos em que a transparência não foi alcançada, foi realizada uma modificação no ajuste do ganho do sistema de frequência modulada e, em seguida, aplicou-se a versão brasileira do “*Phrases in Noise Test*” para avaliar a percepção da fala com ruído competitivo.

**Resultados:**

Observou-se que houve transparência entre o sistema de frequência modulada e o implante coclear em 85% dos participantes avaliados. Depois de ajustar o ganho do receptor do sistema de frequência modulada nos outros participantes, os dispositivos mostraram transparência quando o teste de verificação eletroacústica foi repetido. Observou-se também um melhor desempenho na percepção da fala no ruído após um novo ajuste, ou seja, nesses casos a transparência eletroacústica resultou em transparência comportamental.

**Conclusão:**

O protocolo de avaliação eletroacústica sugerido foi eficaz para avaliar a transparência entre o sistema de frequência modulada e o implante coclear. A realização do ajuste do processador de fala e o ganho do sistema de frequência modulada são essenciais no momento da adaptação deste dispositivo.

## Introduction

From January 1992 to January 2017, 7893 cochlear implant surgeries were carried out in the Unified Health System accredited services in Brazil.^1^ In this time frame, the device manufacturers invested in new technologies in order to provide maximum effectiveness and accessibility to users, such as new signal processing strategies and directional microphone technologies, in an attempt to improve speech perception in noise.^2^, ^3^, ^4^, ^5^ The frequency modulation (FM) system is a device that enables speech perception in noise by reducing the harmful effects of reverberation and the distance between the speaker and the listener.^6^, ^7^ The FM system is currently considered the most effective approach to improve speech recognition in noise in cochlear implant users.^8^

The FM system was introduced in the Unified Health System in Brazil on June 25, 2013, through Ordinance no. 1274^9^ and is intended for individuals between 5 and 18 years of age. Subsequently, the hearing health services accredited by the Unified Health System throughout the country have been prepared to carry out FM fitting.

Guidelines to assist audiologists in the selection, fitting, and verification of the FM system for children requiring hearing aids have been made available.^10^ According to these guidelines, performing a careful check is required before fitting the FM system to hearing-impaired children. The electroacoustic test recommended for hearing aids adopts the concept of transparency between the FM system and the hearing aid.

Although there are recommendations regarding the behavioral tests that should be performed at the fitting the FM system to cochlear implant users,^10^ there are no published recommendations regarding the electroacoustic test. Indeed, there is only one report suggesting the performance of an electroacoustic verification protocol of FM system coupled to the cochlear implant for the assessment of the transparency between the devices.^11^

Considering that each child with a cochlear implant has its own pace of oral language development, which is related to intrinsic and extrinsic factors, a significant number of children may not be able to report the benefit of coupling the FM system to the cochlear implant only through behavioral measures. Thus, it is necessary to implement a thorough protocol that includes objective tests for electroacoustic verification, in order to achieve the best use and to fit the device accurately regardless of the child's response.

By providing this real benefit to the patient, it is expected that the adherence to the use of the FM system will increase. Thus, in addition to the individual benefits, the implementation of a thorough protocol will also have a positive impact on the public policies of hearing health care in Brazil. Indeed, the FM system is an expensive device, and it is thus essential that the hearing health services are enabled to perform accurate fitting in order to avoid negative impacts on the users and the public money.

## Methods

The study was carried out in the Craniofacial Anomalies Rehabilitation Hospital, University of São Paulo, Bauru, São Paulo, Brazil and began once the approval of the Research Ethics Committee was obtained, under opinion no. 1,992,217.

### Case selection

The sample included 40 participants between the ages of 5 and 18 years, who were users of the following speech processors: Harmony (15%), Opus 2 (60%), Freedom (7.5%), and Nucleus 5 (17.5%). The participants were selected according to the demand of attendances of the Craniofacial Anomalies Rehabilitation Hospital in which the research was carried out from November 2015 to August 2016. The researcher responsible for data collection visited the research site during the days and times when there were requests for adaptation of the FM system. Hence, he personally selected the participants for the study and provided clinical care.

The following inclusion criteria were established:

- Children between 5 and 18 years of age;
- Children with established or developing oral language;
- Effective cochlear implant users for at least 3 years;
- No other impairments associated with hearing.

### Speech processor adjustment

For the fitting of the FM system, the speech processors were adjusted in relation to the audio-mixing, following the recommendations of Schafer and Wolfe.^12^

### Adjustment of the FM system

The assessments were performed upon fitting of the FM system, and all participants received the Phonak-branded Inspiro Premium FM transmitter. All the fitted receivers consisted of electrical coupling and varied according to the speech processor model. Users of the Harmony and Opus 2 speech processors received the MLxi universal receiver, which was coupled to the former using the iConnect hook and to the latter using the battery cover with the FM entry. Users of the Freedom and Nucleus 5 speech processors received specific receivers, using the Microlink Freedom model for the former and the ML14i for the latter.

The FM receivers of the 40 participants were adjusted using the Phonak “FM SuccessWare” software, version 4.6.3, which automatically calculates the gain according to the speech processor model. Table 1 summarizes the gain settings used for each speech processor model.

**Table 1:** FM gain recommended for each speech processor model automatically calculated by the Phonak “FM SuccessWare” software, version 4.6.3.

Speech processor model	Brand	FM gain recommended
Harmony	Advanced Bionics	+10 dB
Freedom	Cochlear	0 dB
Nucleus 5	Cochlear	+2 dB
Opus 2	MED-EL	+10 dB

### Electroacoustic evaluation

An electroacoustic evaluation was performed for all 40 participants. For this evaluation, we used the Audioscan Verifit device with the HA-1 coupler and the listening check devices corresponding to each speech processor model. We also used a sound attenuation box made by a foam-lined plastic box.

In this methodology, the electroacoustic verification test was used to evaluate the transparency between the FM system and the cochlear implant as suggested by Schafer et al.^11^ The step-by-step process is depicted in Table 2, Table 3.

**Table 2:** Step 1: step-by-step electroacoustic evaluation test – IC only.

a	Connect the IC speech processor to its respective listening check device
b	Place one side of the earphone (listening check device) on the HA-1 coupler through the use of putty and place them in the Verifit test box
c	In the Verifit choose: “test” – “test box measures” – “speechmap”
d	Place the IC speech processor in the test box with the microphone next to the reference microphone (Verifit)
e	Close the equipment and select the stimulus intensity 65 dB SPL and stimulus type “Speech-ISTS”
f	Writhe down the values obtained in the frequencies of 750, 1000 and 2000 Hz and calculate a mean of these values

**Table 3:** Step 2: step-by-step electroacoustic evaluation test – IC + FM system.

a	Adjust the gain of FM system (company's software)
b	Plug the FM system receiver into listening check device of the speech processor
c	To put the speech processor in the sound attenuation box
d	Put the microphone of the FM system transmitter in the test box (Verifit) near the reference microphone
e	In the Verifit choose: “test” – “test box measures” – “speechmap”
f	Close the equipment and select the stimulus intensity 65 dB SPL and stimulus type “Speech-ISTS”
g	Writhe down the values obtained in the frequencies of 750, 1000 and 2000 Hz and calculate a mean of these values

According to the above-mentioned study, the devices with difference between the means of the evaluated frequencies equal to or less than 3 dB were considered transparent. In cases where the transparency was not achieved, i.e., when the difference between the means was greater than 3 dB, a modification in the FM gain adjustment was made using Phonak “FM SuccessWare.”

### Evaluation of speech perception

The speech perception test was performed in cases where electroacoustic transparency was not achieved and FM system gain adjustment needed to be modified in order to verify if electroacoustic transparency causes behavioral transparency. This was done because there would be no way to otherwise compare two test situations of speech perception, because FM gain adjustment does not need to be modified in cases with transparency between the devices.

The test used to assess speech perception in cases where transparency was not achieved was the “Phrases in Noise Test (PINT) – Brazilian version”^13^ because it is one of the tests indicated for the assessment of speech perception in competitive noise for young children, particularly for children who are still developing their oral language skills.

The Brazilian version of the PINT test,^13^ includes 10 simple sentences related to body parts, presented at a fixed intensity of 60 dB SPL, and with classroom noise intensity varying from 45 to 72 dB SPL in scales of 3 dB and 8 s inter-stimulus interval. Thus, the test starts in descending Signal to Noise Ratio (SNR) from +15 dB to −12 dB, and ends in ascending SNR from −12 dB to +15 dB.

### Statistical analysis

Descriptive statistical analysis was performed for all models and brands of the cochlear implants. The comparison of the results of the electroacoustic evaluation between different brands and models was performed using analysis of variance (ANOVA). Comparison of the electroacoustic evaluation results in the “implant only” and “implant + FM” conditions was performed using the paired *t*-test.

The results of the speech perception test were not analyzed statistically because of the small sample size. Thus, it was impossible to conclude that the difference between the two assessed situations was statistically significant. Thus, we opted for the qualitative analysis of these results.

## Results

Table 4 summarizes the results of the electroacoustic verification procedure (transparency evaluation). We detected transparency between the FM system and the cochlear implant in 34 (85%) of the 40 participants. The results in which no transparency was observed are highlighted in bold in Table 4.

**Table 4:** Results of the electroacoustic evaluation.

Participant	Speech processor model	Mean of CI microphone (dB)	Mean of CI + FM microphone (dB)	Difference (dB)
1	Harmony	74.6	75	0.4
2		79	78	1
3		78.3	80.3	2
4		75	79.3	**4.3**
5		87	85.3	1.7
6		81.3	80.6	0.7

7	Opus 2	72.6	75.6	3
8		67.6	69	1.4
9		71	74.6	**3.6**
10		72	75	3
11		73.3	71.3	2
12		74	73.6	0.4
13		78.6	77.3	1.3
14		75.3	74.4	0.9
15		68.6	64	**4.6**
16		73	76.6	**3.6**
17		64.6	67.3	2.7
18		78.6	82	**3.4**
19		72.6	75.3	2.7
20		66	67.3	1.3
21		77.3	78	0.7
22		74	75	1
23		70.6	72.6	2
24		79.6	82	2.4
25		54	53.6	0.4
26		78	79.6	1.6
27		78.6	80.6	2
28		71.6	74.6	3
29		70.6	71.6	1
30		73	76	3

31	Freedom	75.3	76.6	1.3
32		73.6	76	2.4
33		68.6	75	**6.4**

34	Nucleus 5	71.3	68.6	2.7
35		65.6	67.3	1.7
36		73.3	70.3	3
37		78.6	76.3	2.3
38		69.6	72	2.4
39		70	69.6	0.4
40		84.3	82.6	1.7

CI, cochlear implant; FM, frequency modulation system.

Based on ANOVA, there was no statistically significant difference between the values of transparency (mean) in the different brands (*p* = 0.545) and models (*p* = 0.327).

Table 5 summarizes the results of the cases in which there was no transparency between the devices, and the results after modifying the FM gain adjustment using the “FM SuccessWare” software.

**Table 5:** Description of the results before and after new FM gain adjustment.

P	Initial gain (dB)	Result of the electroacoustic evaluation^a^ (dB)	Speech perception in noise test^a^ (dB)	Gain after adjustment (dB)	Result of the electroacoustic evaluation^b^ (dB)	Speech perception in noise test^b^ (dB)
4	+10	4.3	0	+8	1.7	−1.5
9	+10	3.6	–	–	–	–
15	+10	4.6	–	+12	1.3	–
16	+10	3.6	+6	+8	2	−1.5
18	+10	3.4	0	+8	0.3	−1.5
33	0	6.4	–	–	–	–

P, participant.

a With initial gain.

b After adjustment of gain.

After adjusting the gain of the FM receiver, the devices of participants 4, 15, 16, and 18 exhibited transparency when the electroacoustic verification test was repeated. Furthermore, participants 4, 16, and 18 exhibited a better performance in speech perception in noise after a new adjustment; thus, in these cases, the electroacoustic transparency caused behavioral transparency.

In the case of participant 15, the FM gain was modified and a new electroacoustic verification was performed. However, the speech perception in noise could not be performed owing to the level of language development. Indeed, participant 15 could not perform the Brazilian version of the Hearing in Noise Test (HINT),^14^ which had been suggested in the initial methodology. It should be noted that the evaluation of this participant was performed at the beginning of data collection and, therefore, before sending the amendment to the Research Ethics Committee requesting the inclusion of the PINT test.^13^ in the methodology. Participant 15 did not return to the service until the end of this data acquisition.

In the case of participant 33, for whom there was no electroacoustic transparency of the FM system coupled to the cochlear implant, the modification in the receiver gain adjustment reduced the FM system gain. Participant 33 used the Freedom speech processor, for which the manufacturer of the FM system Phonak recommends that the FM receiver gain be set to 0 dB (Table 4). Therefore, adjusting the FM gain could negatively affect the FM system benefit for this participant.

Participant 9, for whom there was no electroacoustic transparency of the FM system coupled to the cochlear implant, did not remain long enough in the outpatient routine to perform the procedures of FM gain adjustment and speech perception test.

## Discussion

The results shown in Table 4 reflect the importance of performing electroacoustic verification of the FM system coupled to the cochlear implant, considering that transparency was achieved in 85% of the cases. This result can be explained by the fact that the strict adjustment of the speech processors and FM gain was performed thoroughly. Although the FM systems are conceptually simple in their technologies and handling, one major challenge in fitting them is the need to strike a proper balance between the gains of this system and those of the hearing aid.^10^

FM system technology has stimulated the initial questions, for example, whether a monaural fitting is better than a binaural one, and suggested questions related to adjustments performed in the speech processor of the cochlear implant and in the receiver of the FM system during fitting.^15^ A study in adults using unilateral cochlear implants concluded that FM receiver gain has the potential to directly influence speech recognition in noise.^16^ Another study compared speech recognition in noise in adolescent and adult users of FM systems electrically and electromagnetically coupled to cochlear implants. The findings indicated that different FM gain adjustments may be required to achieve optimal performance.^17^ Clinical studies have shown that gain adjustments in wireless communication systems can have a considerable effect on the performance of the users of these devices when coupled to the cochlear implant.^18^

The speech recognition in noise test “Phrases in Noise Test” (PINT)^13^ was performed in three out of the 6 (15%) cases in which transparency was not achieved (Table 4). Table 5 indicates a better performance of the three participants in the PINT^13^ test after the new adjustment of the receiver gain when the devices were set up in order to achieve transparency. Based on this, we can suggest that electroacoustic transparency caused behavioral transparency. These participants needed a new gain setting to obtain greater benefit from the FM system, thus proving that the effective benefit is directly related to an adequate adjustment of the receiver gain, which is in line with previous studies.^11^, ^18^ Considering that these three participants were the youngest in the present study and were developing their language skills, the need for this new adjustment was only realized after the electroacoustic verification test. Thus, it is perceived that the use of a thorough protocol during the fitting of the FM system to the cochlear implant in children, including objective tests in addition to behavioral tests, is essential to ensure effective measurement and fitting.

A previous study evaluated the frequency of use of the FM system in 70 users of cochlear implants in the classroom. A total of 52.8% said they did not use or partially used the device. Thus, although the literature reports the advantages of using the FM system, there are still patients who own the device and do not use it. The authors found that 10% out of the 52.8% of the patients who did not use of the FM system effectively stated that they did not perceive any benefit in the FM system. It can be affirmed that the proper adjustment of the FM system is directly related to the greater benefit of the technology and, therefore, to the greater adherence to its use. Thus, in addition to providing a proper fitting, the electroacoustic verification test, when related to increasing adherence to the use of FM systems, can positively affect the public policies related to granting this device to cochlear implant users, thus potentially avoiding the waste of public funds.^19^

As discussed previously, there is only one previous report on the development of an electroacoustic evaluation protocol for FM systems coupled to cochlear implants.^11^ The authors evaluated transparency in three models of speech processors: Harmony, Opus 2, and Nucleus 5, and made different combinations using Phonak and Oticon FM transmitters. Thus, the discussion of the data shown in Table 1, Table 2 is comparatively based on the results reported in this previous study regarding the use of the Inspiro transmitter and MLxi receptors, both Phonak-branded, which are the same used in our study.

Table 4 indicates that only 1 (16.6%) of the six tests performed on the Harmony speech processor model did not show transparency. It was necessary to adjust the FM gain in the case of participant 4, in which the gain of +10 dB was reduced to +8 dB in order to achieve transparency (Table 5). The above-mentioned study^11^ established combinations among three different receivers and four different transmitters for the test with the same speech processor. The authors reported that, when using the Inspiro transmitter with the MLxi receiver coupled to the Harmony speech processor, transparency was achieved with gain of +4 dB. This variability of the FM gain adjustment to achieve transparency may occur, but the speech perception test must be performed to verify if the benefit is maintained.

For the tests performed with the 24 users of Opus 2 speech processors, there was no transparency in 4 (16.6%) cases. From these, it was possible to perform a new adjustment in the FM gain in three cases. As shown in Table 5, it was necessary to increase the FM gain from +10 dB to +12 dB for participant 15 in order to achieve transparency. For participants 16 and 18, it was necessary to decrease the FM gain from +10 dB to +8 dB. These results are in line with previous data.^11^ The evaluation with the Opus 2 speech processor indicated that variable gain settings were necessary in order to achieve transparency. In particular, in the combination of the MLxi receiver and the Inspiro transmitter, the authors found that the FM gain required to achieve transparency was 0 dB. In 83.4% of the cases that presented transparency without the need of a new adjustment, it was observed that there was a difference of 10 dB between the results found in the present study and the results found in the previous study.^11^

The interest of this study lies in the fact that the results indicated a variability in the FM gain adjustment when the same speech processor model was coupled to the same FM system technology, but with different users (*n* = 33), except for the Nucleus 5 speech processor model. This is in contrast to the findings reported in the United States,^11^ which pointed to variability in the FM gain adjustment when a single speech processor was coupled to different FM system technologies.

Regarding the electroacoustic evaluation of the FM system coupled to the Nucleus 5 speech processor, Table 4 shows that transparency was achieved in the seven (100%) cases with FM gain at +2 dB, with no need for a new adjustment. The gain settings that are needed to achieve transparency have been previously reported to be highly variable in a study combining four transmitters and four receivers for the evaluation of transparency with the Nucleus 5 speech processor.^11^ Regarding the use of the Inspiro transmitter combined with the use of the Phonak receivers, the authors reported that it was not possible to achieve transparency when using the MLxS receiver, a previous generation receiver. For the use of the MLxi receiver, it was necessary to adjust the FM gain to +8 dB for transparency. Comparing the results obtained in the present study with those obtained in the aforementioned study, a 6 dB difference was observed in the gain needed to achieve transparency. However, it is noteworthy that this study used the ML14i receiver, a specific device for connection to the Nucleus 5 speech processor. Thus, the difference between the gain settings can be explained by the fact that those authors used a universal receiver, which requires an adapter to be connected to the cochlear implant. Thus, the receivers used differed in the connection mode. In this study, all FM receivers coupled to the Nucleus 5 speech processor belonged to specific models.

In summary, owing to the variability found in the FM gain settings to achieve transparency, it is highly recommended to perform the electroacoustic verification test of the FM system when coupled to the cochlear implant, particularly in children. It is believed that the transparency between the FM system and the cochlear implant can positively affect the adherence to the use of the FM system, and will consequently affect the best use of public money in relation to the granting of this device by the Brazilian Unified Health System. Indeed, between 2013 and 2017, 16,141 FM system Kits were granted under Ordinance 1274.^9^ The longitudinal monitoring of these participants in future studies may provide more specific information about their adherence to the use of the FM system.

## Conclusions

Based on the present findings, we conclude the following: (1) The electroacoustic evaluation protocol suggested was effective in evaluating transparency between the FM system and the different models of speech processors of cochlear implants; (2) There was a greater benefit in the speech recognition in noise when there was a transparency between the FM system and the cochlear implant; (3) Adjusting the FM system gain can be very helpful for many cases when fitting this device.

## Funding

The research reported in this publication was supported by the Coordination of Superior Level Staff Improvement (10.13039/501100002322CAPES) and by São Paulo Research Foundation (10.13039/501100001807FAPESP) grant no. 2014/20398-1.

## Conflicts of interest

The authors declare no conflicts of interest.
